# Digital stressors and resources perceived by emergency physicians and associations to their digital stress perception, mental health, job satisfaction and work engagement

**DOI:** 10.1186/s12873-024-00950-x

**Published:** 2024-02-27

**Authors:** Monika Bernburg, Anika Tell, David A. Groneberg, Stefanie Mache

**Affiliations:** 1 Institute of Occupational, Social and Environmental Medicine, Goethe University, Frankfurt, Germany; 2 Institute for Occupational and Maritime Medicine (ZfAM), University Medical Center Hamburg- Eppendorf (UKE), Seewartenstraße 10, 20459 Hamburg, Germany

**Keywords:** Digitization, Documentation technologies, Electronic health records, Emergency medicine, Hospital staff, Medical staff, Physicians, Preventive measures, Technostress

## Abstract

**Background:**

Digital technologies are increasingly being integrated into healthcare settings, including emergency departments, with the potential to improve efficiency and patient care. Although digitalisation promises many benefits, the use of digital technologies can also introduce new stressors and challenges among medical staff, which may result in the development of various negative work and health outcomes. Therefore, this study aims to identify existing digital stressors and resources among emergency physicians, examine associations with various work- and health-related parameters, and finally identify the potential need for preventive measures.

**Methods:**

In this quantitative cross-sectional study, an online questionnaire was used to examine the relationship between digital stressors (technostress creators), digital resources (technostress inhibitors), technostress perception as well as mental health, job satisfaction and work engagement among 204 physicians working in German emergency medicine departments. Data collection lasted from December 2022 to April 2023. Validated scales were used for the questionnaire (e.g. “Technostress”-scale and the Copenhagen Psychosocial Questionnaire (COPSOQ). Descriptive and multiple regression analyses were run to test explorative assumptions.

**Results:**

The study found medium levels of technostress perception among the participating emergency physicians as well as low levels of persisting technostress inhibitors. The queried physicians on average reported medium levels of exhaustion symptoms, high levels of work engagement and job satisfaction. Significant associations between digital stressors and work- as well as health-related outcomes were analyzed.

**Conclusion:**

This study provides a preliminary assessment of the persistence of digital stressors, digital resources and technostress levels, and their potential impact on relevant health and work-related outcomes, among physicians working in German emergency departments. Understanding and mitigating these stressors is essential to promote the well-being of physicians and ensure optimal patient care. As digitisation processes will continue to increase, the need for preventive support measures in dealing with technology stressors is obvious and should be expanded accordingly in the clinics. By integrating such support into everyday hospital life, medical staff in emergency departments can better focus on patient care and mitigate potential stress factors associated with digital technologies.

**Supplementary Information:**

The online version contains supplementary material available at 10.1186/s12873-024-00950-x.

## Introduction

Digitalization in emergency care refers to the use of digital technologies and tools to improve the delivery of emergency medical services [1]. In the medical discipline of emergency medicine there are many possible and promising applications of digital technologies or opportunities for digitising processes, such as the introduction of hybrid examination rooms with live communication possibilities with external medical experts, or the use of mobile technologies to facilitate communication between healthcare providers, emergency data management (i.e. digtial bedside cards) or clinical decision support systems [2–4].

The use of information and communication technologies (ICT) in hospitals in general has many benefits, which are now widely recognised and have been investigated in numerous studies. Research suggests that ICT can improve the traceability of documentation because patient data can be entered and accessed much more quickly and easily, or it can improve work processes and communication between medical and nursing staff [5].

One of the most visible manifestations of digitisation in healthcare today is the introduction of the electronic health record (EHR), which collects a wide range of patient health data, such as details of diagnoses, therapies or medications, and provides a transparent and constantly updated digital overview of patient data for medical staff and other healthcare professionals [6, 7].The use of electronic medical records (EMRs) in emergency care has been explored in several studies: all all emphasise the importance of seamless and workflow-based EMRs in managing emergency patients [8, 9].- However, Yamamoto (2006) highlights the challenges of EMR implementation in the emergency department, including the unique demands of this setting and the need to carefully consider the advantages and disadvantages of computerized charting [10].

Digital tools also enhance communication among healthcare providers involved in emergency care. For example, secure messaging platforms allow for quick exchange of information between paramedics, emergency room staff, and specialists [11].

Digital devices can be used to remotely monitor vital signs and other health parameters of patients during emergencies [12]. This helps in early detection of deteriorating conditions and enables timely interventions.

Digitalization supports the development and implementation of decision support systems that provide evidence-based guidelines and recommendations to healthcare providers during emergencies. These systems help ensure standardized care practices and improve patient outcomes [13].

Overall, digitalization in emergency care has the potential to enhance efficiency, accuracy, and timeliness in delivering emergency medical services. It can improve patient outcomes, reduce errors, and enable better coordination among healthcare providers involved in emergency response. So, further use of digital technologies will make the provision of care more transparent, effective and efficient [14, 15].

However, in addition to the benefits mentioned above, the introduction of digital tools and ICT as well as the resulting changes can also be associated with a number of drawbacks. The use of ICT has the potential to cause stress and strain among health professionals due to a lack of usability, the high cost of the technologies, the length of implementation projects, or the data security issues that need to be addressed in the implementation process [16].

In this way, emergency physicians face numerous digital stressors in their daily work, including the overwhelming amount of communication, documentation and patient information. These digital stressors can contribute to a high cognitive load and increase the risk of information overload for emergency physicians. Having to constantly juggle and prioritize the influx of messages and notifications can lead to heightened stress levels and decreased efficiency in managing patient care [17].

Therefore, it is becoming increasingly important to consider the impact of hospital digitisation processes on the health of medical staff, and to learn more about how relevant stressors and resources interact in this setting.

### Theoretical framework

The Technostress model and the Job Demands-Resources (JD-R) model were used in this study to better examine and understand the interplay between influencing factors, digital stress and related mental health outcomes [18, 19].

**Fig. 1:**
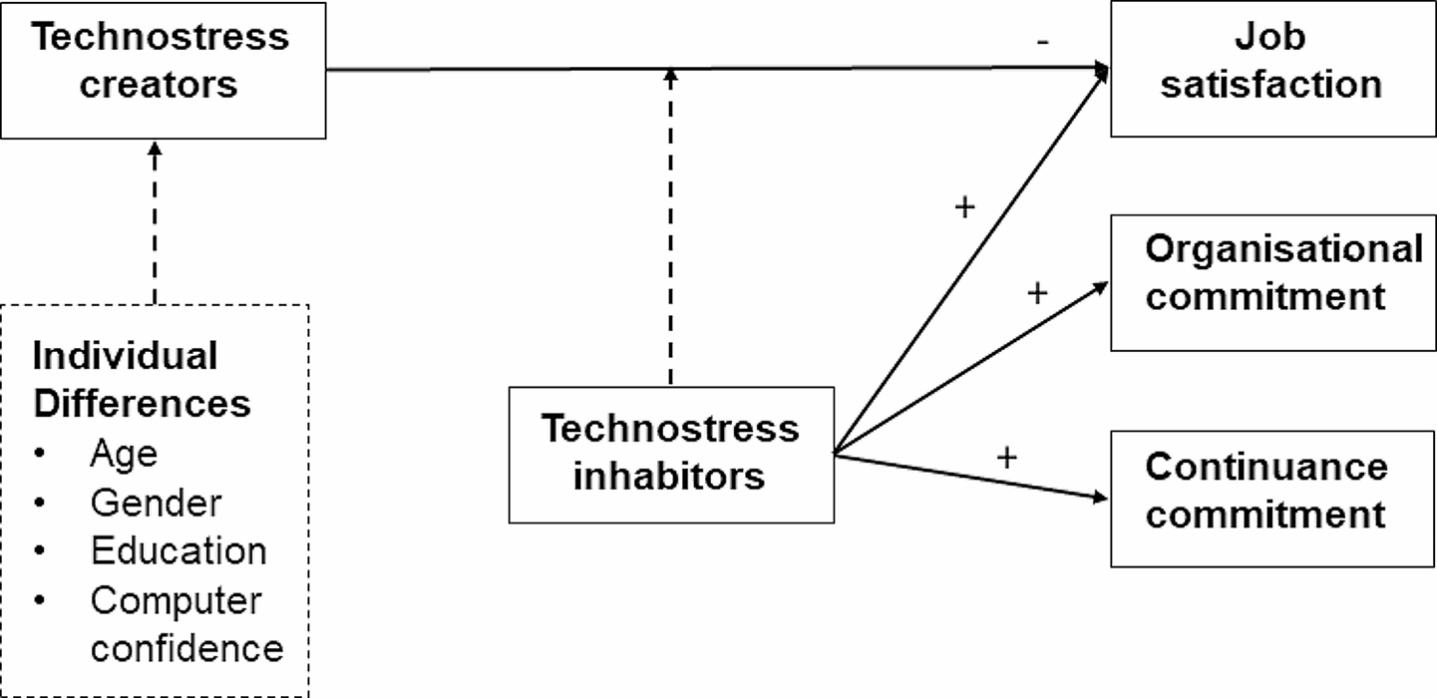
Technostress creators and technostress inhabitors (Ragu-Nathan et al. 2008)

According to Ragu-Nathan et al. “the stress experienced by end-users in organisations as a result of their use of ICT” is known as technostress [20]. Technostress creators, also known as digital stressors, and technostress inhibitors, also known as digital resources or protective factors, were the two main constructs developed and empirically validated by Ragu-Nathan et al. [20]. The researchers found the following five factors within the Technostress creators construct: “techno overload”, “techno invasion”, “techno complexity”, “techno insecurity” and “techno uncertainty”. Technostress inhibitors are the factors, strategies, and practices that individuals and organizations can employ to mitigate the negative effects of technostress. By understanding and implementing these inhibitors, individuals can better manage their digital habits, maintain a healthy work-life balance, and reduce the impact of technostress on their overall well-being. Authors identified the ‘technostress inhibitor’ factors of ‘literacy facilitation’, ‘technical support provision’ and ‘involvement facilitation’ [20]. Figure 1 illustrates the technostress model for a better understanding.

### Current state of research

The current scientific evidence on technostress in general has shown higher levels of stress among employees working with digital technologies and has identified a variety of technostress promoters as well as protective factors [21]. The aforementioned studies on the persistence of technostress in the health sector have provided a first impression of the prevalence of technostress among medical staff, as well as the persistent stressors, resources and outcomes related to digitalisation [22].

According to a recent study clinicians who regularly used electronic health records (EHRs) reported experiencing digital stress [23]. These findings are supported by a subsequent study, which assessed the digital stress levels of medical staff working in hospitals [22]. The study found moderate levels of stress in the study group, while nurses and doctors reported high levels of stress. Conversely, higher levels of social support appeared to reduce technostress and were strongly inversely related to it [22].

Further specific stressors associated with EHRs are inadequate instruction on how to use the technology, less face-to-face time with patients, too much time spent on data entry, and a general increase in computerisation at work [24].

However, research on digital stress in hospitals, particularly among the group of doctors working in emergency departments, is still in its early stages.

### Objectives

**Fig. 2:**
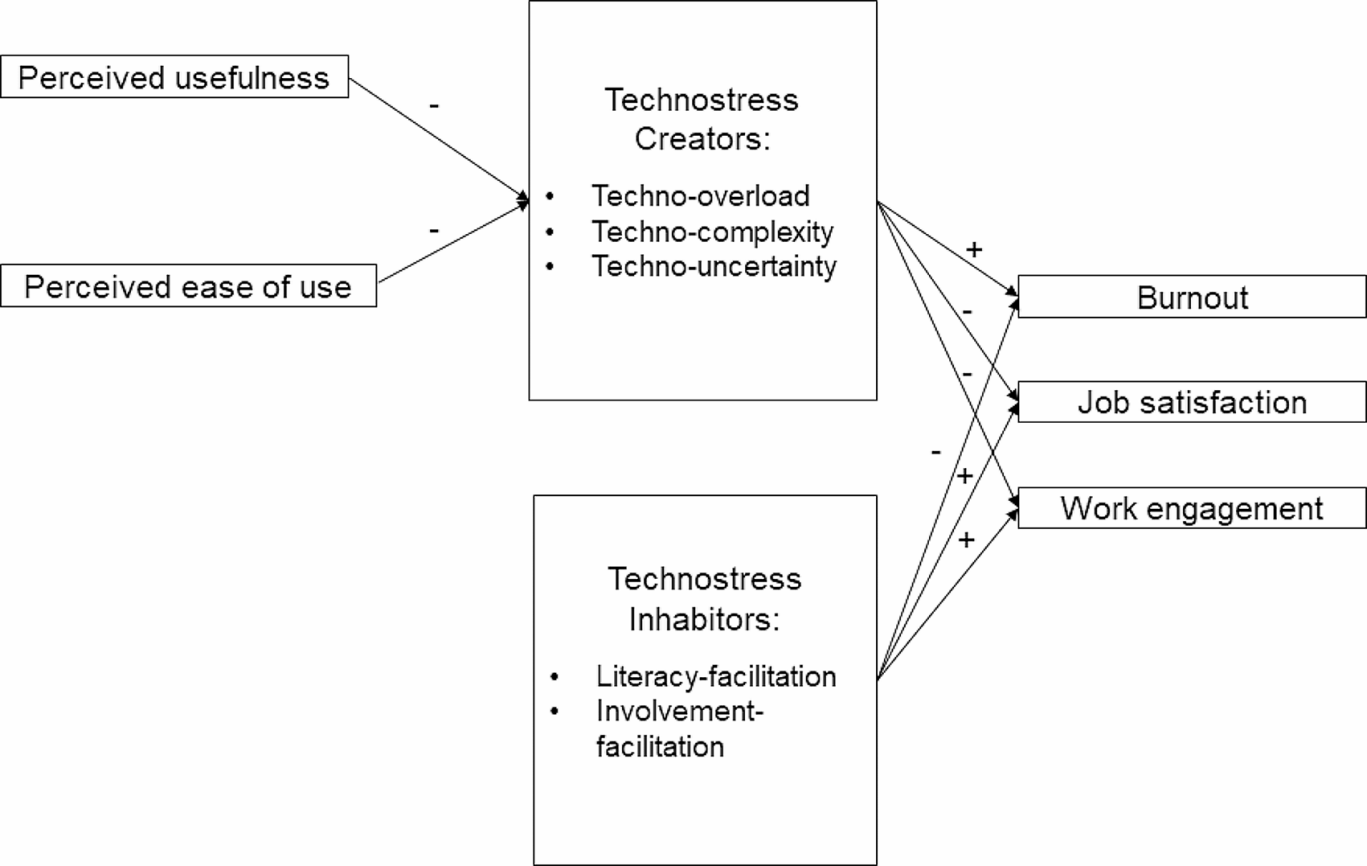
Conceptional model

The aim of this study is to conduct a quantitative study to (1) analyse the relationships between digitalisation processes and the daily activities of medical staff working in emergency medicine hospital departments, (2) identify the stressors and resources arising from the use of digital technologies in emergency medicine.In addition, the relationship with mental health and work-related outcomes (4) and the need for preventive measures (5) will be explored, thus contributing to filling the data gap. A conceptional model for understanding the research context is illustrated in Fig. 2.

The following assumptions have been developed in the light of this information:

#### Assumption 1

Lower levels of the subjectively perceived usefulness and the perceived ease-of-use of the utilized digital technologies are significantly associated with higher levels of Technostress creators and higher levels of perceived Technostress by emergency physicians.

#### Assumption 2

Higher levels of Technostress creators respectively higher levels of perceived Technostress perceived by emergency physicians are.

- significantly related with higher rates of their exhaustion-symptoms,
- significantly related with lower levels of their job satisfaction,
- significantly related with lower levels of their work engagement.

#### Assumption 3

A higher perception of the Technostress inhibitors among emergency physicians is.

- associated significantly with lower rates of exhaustion-symptoms,
- associated significantly with higher levels of job satisfaction,
- associated significantly with a higher-rated work engagement.

#### Assumption 4

Technostress-levels are significantly lower in those emergency physicians whose employers already offer preventive measures (e.g. information or qualifications), to a higher degree.

## Materials and methods

### Study design and sample characteristics

This quantitative study was conducted in form of a cross-sectional, online-based questionnaire survey in emergency medicine hospital departments in Germany. Working as a physician in an emergency hospital department was an eligibility criteria for the study participants. As further criteria, it was defined that the study participants must utilize digital technologies for clinical documentation purposes, such as the EHR or special documentation software at least once a week, meaning consequently of course that the emergency hospital department must have implemented at least one of such digital technologies.

A minimum total sample size of *n* = 200 emergency physicians was targeted, calculated by using G*Power in the version 3.1.9.6 and by assuming an a priori power-analysis with alpha = 0,05, 95% confidence intervals and a medium effect size for all planned analyses.

### Data collection

The online survey was conducted within a period of roughly two months from end of December 2022. The corresponding relevant emergency hospital departments were identified with the results of several internet portals. The study participants were then initially recruited via E-mail-contact or directly via telephone. After 3 weeks reminders where send to all physicians.

### Measures

Based on the theoretical background, technostress creators were assessed as job demands and technostress inhibitors as job resources (independent variables). We assessed three outcome variables: burnout, job satisfaction, work engagement. Additional file 1 provides an overview of the main variables and their measurement.

#### Sociodemographic and work-related variables

In the first part of the questionnaire, sociodemographic data were collected including information on the job position, utilization of digital documentation technologies, age, sex, regional structure and ownership of the clinic, duration of occupation in the respective emergency department and work experience in the field in general as well as weekly working hours were queried first.

#### Usage frequency and attitudes regarding digital technologies

The frequency and duration of utilization of the digital documentation technologies as well as the attitude towards the technologies were measured. For the assessment of utilization frequency and duration two self-developed items were utilized. Additionally, for the query of the attitudes towards the utilized technologies the two validated construct-scales “Perceived Usefulness” (PU) and “Perceived Ease of Use” (PEOU) from the German version of the Technology Acceptance Model (TAM) were used [25]. Cronbach’s alpha- values were at 0.85 for the PU scale and at 0.79 for the PEOU scale, thus indicating good to almost very good reliability.

#### Technostress creators and technology-associated resources

In the second thematic part the persistence of digital stressors in the workplace is measured. For this the standardized and validated “Technostress”-scale by Ragu-Nathan et al. (2008) was used in an adapted version including “Technostress”-creators “Techno-overload”, “Techno-complexity” and “Techno-uncertainty” in the German version [21]. This instrument has an acceptable to good reliability with Cronbach alpha-values for the different constructs and good discriminant and convergent validities with no significant error correlations between the items [20]. Additionally, for a more specific query of the stressors, a self-developed item was utilized based on an item from the HIMSS-study (2015) [16]. To get an overview about the persisting protective factors (resources) the two Technostress-inhibitor-constructs “Literacy facilitation” and “Involvement facilitation” from the Technostress-scale by Ragu-Nathan et al. (2008) were used with a total of 9 items [20].

#### Preventive measures

The third thematic block covered several items for the query of preventive measures with two constructs, [26], assessing the already implemented preventive measures with Likert-scale- (8 items in total) [27] These items were further complemented by a self-developed scale querying the benefit of the already implemented preventive measures as well as by three additional self-developed items in free text format intended to capture positive and negative aspects of the preventive measures as well as need for further preventive measures.

The preventive measures variables were divided into three groups: “disagree” (corresponding to a low degree of precautions implemented and “partially agree” (corresponding to an average level of protective measures protection) and “agree” (corresponding to a high degree of precautions implemented.

#### Work- and mental health-related outcomes

Next, several health- and work-related outcomes were assessed in another thematic block, all by utilizing standardized and validated scales. The outcome “burnout-symptoms” was measured with the homonymous standardized and validated scale from the COPSOQ (2022), which consists of 3 items [28]. Further, the outcome “job satisfaction” was measured with the 3-item-construct by Ragu-Nathan et al. (2008) [20]. In the analysis the COPSOQ-scale “burnout-symptoms” showed a good reliability with a Cronbach’s alpha-value of 0.81. For the utilized “job satisfaction”-scale, the reliability showed to be also high with a Cronbach’s alpha-value of 0.85.

Using another scale of the COPSOQ, we evaluated employee engagement at work. There are 3 items total in this self-report questionnaire [28].

Previous research examined psychometric data and confirmed the scale’s validity and reliability. Cronbach’s alpha was also tested, and the result was 0.82.

#### Statistical data analysis

Data was checked for missing values and plausibility. We used 95% confidence intervals or an α-level of ≤ 0.05 for significance tests. Correlation analyses (Pearson’s correlation coefficient for continuous variables, Spearman’s Rho correlation coefficient for ordinal variables) were used. If the requirement of normal distribution of the continuous variables was not fulfilled, the correlations were analysed by using the bootstrapping-method. In addition, multiple regression analyses were applied, controlling for potential confounding variables. Non-parametric tests (Chi^2^- test, Mann-Whitney U- test, Kruskal Wallis- test) were caried out for group differences. In addition, appropriate parametric test procedures (t-test, ANOVA) were applied after testing for normal distribution of the variable-data. We used the statistical software IBM SPSS in the version 27.

## Results

### Sample description

A total of *n* = 251 physicians working in emergency medicine hospital departments took part in the online survey. After checking for missing values and plausibility, 47 questionnaires had to be excluded. Most of the participating physicians were male (57.4%; *n* = 204) (see Table 1).

Furthermore, 27.5% were employed as senior physicians (*n* = 204), 59.5% had been employed in their clinic for more than 4 years (*n* = 204), and in general, mostly had been working in the clinical field for more than 25 years already (31.9%; *n* = 204). Regarding the usage of digital documentation technologies, the electronic health record (EHR) was the most frequently chosen answer category (85%; *n* = 204), followed by the options of additional software (84%) and additional digital devices or hardware (62%).

**Table 1:** Characteristics of study population and hospitals (*n* = 204)

Characteristic	Frequency (n)	Percentage (%)	
*Gender*			
Male	117	57	
Female	87	43	
*Age*			
20–29 years	29	14.3	
30–39 years	62	30.4	
40–49 years	50	24.5	
50–59 years	39	19.1	
60 years and older	24	11.7	
*Job position*			
Assistant physician	51	25.0	
Specialist physician	58	28.4	
Senior physician	56	27.5	
Head physician	39	19.1	
*Extent of current employment*			
Working full time (≥ 35 h/week)	171	83.8	
Working part time (15–34 h/week)	33	16.2	
*Duration of employment with employer*			
< 1 years	12	6.1	
1–<2 years	18	8.7	
2–<3 years	22	10.9	
3–<4 years	31	14.8	
≥ 4 years	121	59.5	
*Overall clinical experience*			
< 5 years	8	4.1	
5–<10 years	26	12.8	
10–<15 years	33	16.1	
15–<20 years	38	18.7	
20–<25 years	34	16.4	
≥ 25 years	65	31.9	

### Descriptive statistical analysis

#### Digital documentation technologies’ frequency and duration of use

Nearly all of the participating doctors said they would use the digital documentation technologies every day when asked about their frequency of use (98.5%; *n* = 204) (see Table 2).

**Table 2:** Frequency and duration of usage of digital documentation technologies (*n* = 204)

Characteristic	Frequency (n)	Percentage (%)
*Usage frequency*		
Daily usage	201	98.5
Usage several times per week	3	1.5
*Usage duration (estimated per day)*		
< 1 h	4	1.8
1–<2 h	51	24.6
2–<3 h	69	38.6
3–<4 h	37	19.3
4–<5 h	23	9.6
5 h or more	12	6.1

#### Perceptions of technostress creators in emergency care

In general, the measured average technostress level of all participants was at a medium level, with a mean of the three technostress-creators of M = 3.18 (1 = do not agree at all/ no technostress; 5 = fully agree/high technostress levels) and an SD = 0.69. Regarding the single technostress creators, the highest mean was observed for the construct of techno-overload (M = 3.52; SD = 0.81), techno-complexity (M = 3.01, SD = 0.75) and techno-uncertainty (M = 2.89, SD = 0.82).

Additionally, a number of potentially harmful side effects or stressful elements were investigated. The aspect of double documentation was rated as the most stressful by 19.5% of physicians (*n* = 204), followed by technical system errors with a share of 17.1%, the control tool for health insurances (12.9% of physicians), and a lack of PC workstations with 15.2%. More than half of the participants reported feeling stressed by the double documentation aspect of technology frequently or very frequently. As 55.2% of the participants said, they were either never stressed by it or only very rarely. The lack of data security did not appear to be seen as a problem.

#### Technostress inhibitors and resources

A low to moderate level of persistent resources was indicated by the mean of the overall expression of technostress inhibitors, which was calculated as M = 2.71 (SD = 0.82). While the level of the technostress inhibitor of literacy facilitation was moderate (M = 3.1; SD = 1.05), the inhibitor of involvement facilitation was particularly low, with a mean of only M = 1.91 (SD = 0.9), indicating that this inhibitor was not perceived as a powerful resource.

#### Prevention measures in the use of digital technologies

Prevention measures and actions were generally only applied infrequently or to a moderate extent (M = 2.32; SD = 0.81). The most frequently used preventive measures were qualifications, with doctors reporting a mean of 3.01 (SD = 1.02) that they received an additional qualification if necessary or that they were adequately qualified during the introduction of new technologies. Additionally, 68% of the participants (*n* = 204) said that their employer would provide enough end devices. However, only 24% of the participants reported that their employer had thought about implementing end devices that wouldn’t obstruct the doctor-patient conversation. Only 19% of the doctors claimed that their employer had planned the technology rollout only after ensuring the system’s stability in order to lessen time-consuming double documentation. Nearly 68% (*n* = 204) of the doctors agreed or strongly agreed that the preventive measures already in place had been beneficial. Only 49% of the participants were very satisfied, and more than 42% of the doctors also said they were not satisfied or not at all satisfied with the preventive measures that had been put in place.

#### Technology acceptance in emergency care

The assessment of technology acceptance showed a mean of M = 3.59 (SD = 0.87, *n* = 204) for the construct of perceived usefulness, indicating that, on average, physicians agreed with the items or were neutral, corresponding to a generally positive perceived view on the utilized technologies. Regarding the construct scale of perceived ease of use, the query showed an overall mean of M = 3.29 (SD = 0.71, *n* = 204), slightly lower than the first construct scale, indicating that the average physician neither perceived the utilized technologies as easy to use nor as not easy to use.

#### Work- and mental health-related outcomes

Since the average physician only occasionally experienced burnout symptoms (M = 2.89; SD = 0.95; *n* = 204), the persistence of burnout symptoms was generally low. The majority of doctors, on average, reported being satisfied with their jobs, with a mean satisfaction level of 4.14 (SD = 0.71). Work engagement was rated with a mean of M = 4.38; SD = 0.91.

### Analytical statistical analysis

The analyses conducted to test our first assumption revealed a moderately negative correlation (*r* = -0.347; *p* = 0.01); and a moderately negative correlation (*r* = -0.473; *p* = 0.01) between the variables of subjectively perceived usefulness (PU) and technostress (PEOU)(see Table 3). Therefore, these results are consistent with assumption 1.

**Table 3:** Pearson correlation coefficients for technostress, PU and PEOU (*n* = 204)

				Overall Expression of Technostress creators	Perceived Usefulness (PU)	Perceived Ease of Use (PEOU)
Overall expression of technostress creators		Pearson correlation		1	−0.347 **	−0.473 **
		Sig. (2-tailed)			0.000	0.000
		N		204	204	204
	Bootstrap ^1^	Bias		0	−0.005	0.000
		Std. Error		0	0.079	0.081
		95% Confidence Interval	Lower		−0.563	−0.609
			Upper		−0.203	−0.275

**Correlation is significant at the 0.01 level (2-tailed)

^1^Unless otherwise noted, bootstrap results are based on 1000 bootstrap samples

According to the results of the multiple regression analysis, the two independent variables of subjectively perceived usefulness (PU) and subjectively perceived ease of use (PEOU), respectively, could account for 23% of the variance of overall expression of techno-stressors R2 = 0.23 (*n* = 204, *p* < 0.001).

The combined influence of the two independent variables was also significant. The coefficient values for the variables of perceived usefulness and perceived ease of use were b = 0.21 (*p* < 0.05) and b = 0.35, respectively. This indicates that as the variables of PU and PEOU of the corresponding digital technologies increased, the employees’ levels of technostress decreased.

The variables of technostress and burnout symptoms showed a slight positive correlation, *r* = 0.31 (CI: 0.01, 0.41), according to the correlation analyses, supporting assumption 2a,c. < 0.05, and in a small negative correlation, *r* = − 0.29 (CI: −0.497, 0.081) with *p* < 0.05 for the variables of technostress and work engagement. For the variables of overall technostress and job satisfaction, we found a small negative correlation too, with *r* = − 0.22 (CI: −0.52, 0.09), which, however, was significant (*p* < 0.05).Thus, assumption H2b can be verified.

The multiple regression analysis for the technostress creators variables and the outcome burnout revealed that the three independent variables of techno-overload, techno-complexity, and techno-uncertainty, respectively, could account for 14% percent of the variance of the dependent variable burnout in this model. Additionally, the findings were highly significant (p. < 0.001). The influence of the predictor of techno-overload was highly significant (*p* < 0.001). The regression results of the two other predictors, however, were not significant (*p* > 0,05). 9% of the variance of job satisfaction, could be explained by the three independent variables of techno-overload, techno-complexity, and techno-uncertainty by this model (*p* < 0.05). However, only the influence of the predictor of techno-overload was significant (*p* < 0.05). The regression results of the other two predictors, however, were not significant (*p* > 0.05) (see Table 4). The analysis for work engagement showed no significant associations.

**Table 4:** Multiple regression analyses of techno-overload, -complexity, -uncertainty and the outcome variables of burnout, job satisfaction and work engagement (*n* = 204)

Predictors	b ^a^	SE ^a^	t		p
	**Outcome of Burnout Symptoms**				
Techno-overload	0.561	0.128	4.527		< 0.001
Techno-complexity	−0.221	0.093	−0.594		> 0.05
Techno-uncertainty	−0.131	0.054	−1.298		> 0.05
	Notation. R^2^ = 0.141 (*n* = 204, *p* < 0.001).				
	**Outcome of Job Satisfaction**				
Techno-overload	−0.158	0.064	−2.398		< 0.05
Techno-complexity	−0.052	0.070	−1.142		> 0.05
Techno-uncertainty	0.075	0.059	1.628		> 0.05
	Notation. R^2^ = 0.088 (*n* = 204, *p* < 0.05).				
	**Outcome of Work Engagement**				
Techno-overload	−0.137	0.085	2.028		> 0.05
Techno-complexity	−0.082	0.069	−1.157		> 0.05
Techno-uncertainty	−0.079	0.071	−1.358	>0.05	
	Notation. R^2^ = 0.068 (*n* = 204, *p* > 0.05).				

^a^Confidence intervals und standard errors per BCa bootstrapping with 1000 BCa samples

The correlation analyses of the two technostress inhibitor variables “literacy facilitation” and “involvement facilitation” and the burnout variable showed a non-significant association, with *p* > 0.05.

The analyses for the technostress inhibitors and the variable of work engagement resulted in a small positive, *r* = 0.157, non significant result, with *p* > 0.05. We also found a positive, but non- significant association between the two technostress inhibitor variables and job satisfaction (*r* = 0.143; *p* > 0.05. Thus, assumptions 3a, b, c cannot be verified.

To test assumption 4, we conducted one-way ANOVAs for the precautions and variables described above. Levels of digital stress differed statistically significantly between the different prevention groups. F(df: 2, 235) = 2.284, *p* < 0.05. Eta-squared values ​​(n) were 5.325 (between groups) and 71.34 (overall) = 0.08, corresponding to intermediate effects according to Cohen’s rule. This result supports assumption 4.

## Discussion

This study, the first of its kind to our knowledge, was conducted to learn more about the digital stressors and resources of German doctors working in hospital emergency departments. Investigating potential relationships between important health and work-related outcomes and identifying potential needs for preventive measures were key objectives of the study.

### Technology acceptance in emergency medicine care

The results of the perceived usability construct showed that, on average, the physicians surveyed were accepting the digital technologies used, with the majority either slightly accepting or neutral about the usability of the technology. However, the construct of perceived ease of use had a slightly lower mean, corresponding to a slightly less favourable view of the ease of use of the relevant technologies. This suggests that emergency doctors may also experience problems when using digital technologies during work. These findings are consistent with general conclusions about how employees perceive digital technology or about potential barriers to using digital technology [29, 30].

Barriers include the need for evidence-based evaluation, the risk of security breaches and misinformation, interoperability, physician resistance and concerns about data quality, privacy, and regulation [31, 32]. Additionally, the technically-driven development of e-health and telemedicine, lack of common platforms and connectivity, and issues with privacy and data protection pose further challenges [33]. These barriers highlight the need for careful consideration and regulation in the integration of digital technology in medicine care.

### Technology-associated stressors and resources

According to the data presented, the surveyed emergency physicians reported average levels of medium technostress, with the highest levels for the technostress creator of techno-overload, which correlates with a general perception among users of being forced by technology to work faster.

Despite these challenges, it is important to note that the experience of digital stress is only moderately pronounced among emergency physicians. This may indicate that while the use of digital technologies does bring about challenges, it is not a pervasive issue that significantly hinders the ability of emergency physicians to provide efficient and effective care to patients.

As there are currently no other studies in the context of the digital stress experience in emergency medicine, the data is difficult to categorise (see limitations). However, if you look at data from other medical fields [22] you will find a corresponding consistency. However, as the field of work activity/ working conditions and the type of use of digital technologies differ to some extent, comparisons are only meaningful to a limited extent.

The findings are also consistent with those of other recent studies conducted in the medical field and among physicians who experienced particularly high levels of technological overload [34–36]. In the study by Liu physicians showed low levels of technostress. Here, perceived technology dependency, and complexity significantly affect physician technostress when using mobile electronic medical records, whereas perceived usefulness and reliability do not [34]. Heponimie et al. showed that high levels of technical problems and number of systems in daily use were associated with high stress; user-friendliness, perceived benefits, and support for feedback were associated with low stress; experienced users had low stress levels [35].

In general, it is clear that both positive and negative aspects of the technostress experience were presented in this study, so that it must be viewed and evaluated in a differentiated and holistic way. In line, a series of studies have explored the concept of technostress in healthcare IT, highlighting both its positive and negative aspects.

Califf et al. (2015) introduced the idea of techno-eustress and techno-distress, emphasizing the need to differentiate and consider both when studying technostress (see research implications) [37].

When asked about stress perception, emergency physicians cited a variety of digital stressors. These findings are consistent with other research suggesting that technical problems and limitations with the technologies, a lack of technical expertise on the part of physicians, a lack of training, expertise and technical support from the IT service, or a lack of time to implement new technologies and usage in daily work routine are significant barriers to technology adoption [38].

Our findings also show that the majority of doctors surveyed did not have the necessary resources to compensate for possible disadvantages of technologies. Meanwhile, moderate levels were still measured for the resource of literacy facilitation, which corresponds to the provision of the necessary training, qualifications and information. Levels were particularly low for the resource of facilitating participation. These results are consistent with recent academic research showing a lack of participation resources, which is consistent with our participation facilitation construct [30].

Relevant resources included peer support, individual resources, such as digital literacy or learning by doing, and organisational resources, such as effective IT support, operational back-up procedures or administrative support [34]. A further study also identified transparency, high quality and sufficient training, availability of technology vendors for questions or problems, coaching and peer monitoring as other important resources [39]. The provision of internal technical support is an important factor [40]. Bregenzer et al. (2021) again emphasise the importance of leadership and identify a health-promoting leadership style as another critical resource [41]. In addition to technical resources or stress inhibitors, coping mechanisms and coping styles can also be used to reduce the manifestation of negative mental health outcomes [42].

### Associations among technostress creators and technostress inhibitors and burnout, job satisfaction and work engagement

Our results indicate a positive correlation between technostresscreators and the perception of burnout symptoms. These findings are consistent with recent research, for example, that physicians surveyed believed that EHR-related stressors contributed to their burnout symptoms [23].

The study found that techno-stressor and psychological capital significantly impact burnout and task performance, highlighting the importance of managing these factors to improve employee task performance and reduce the risk of burnout [43]. Maier et al. (2018) further supported this, demonstrating that technostress can lead to burnout, which in turn decreases employee performance [44].

As there was a significant correlation between technostress and the resulting level of job satisfaction, assumption 2 could also be supported. This is consistent with recent studies that have found significant negative correlations between job satisfaction outcomes and different techno-stressors or overall levels of techno-stress [45].

Several studies have found a significant negative correlation between technostress and job satisfaction. LaTorre (2020) and Marchiori (2019) identified a strong influence of technostress creators on job satisfaction, with factors such as techno-overload, techno-insecurity, techno-uncertainty, techno-complexity, and techno-invasion being particularly impactful [46, 47].

Tarafdar (2019) and Califf et al. (2015) revealed that techno-eustress and techno-distress can significantly impact job satisfaction and turnover intention in different ways. Techno-eustress has a positive effect on job satisfaction only, and techno-distress has a negative effect on job satisfaction and turnover intention [37, 48]. This framework challenges the traditional view of technostress as purely negative, suggesting that it can also lead to positive outcomes [48].

Interestingly, there was no significant associations between the technostress creators and the level of emergency physicians’ work engagement. There is a lack of comparative studies from the medical care context to correlate this result. Other studies show significant correlations with work engagement. Here, research shows a positive association between technostress and work engagement [49, 50]. However, Mohammed [51] found no significant relationship between technostress and work engagement or perceived supervisor support, contrary to existing literature [51]. They conclude, the impact of technostress on work engagement may vary depending on factors such as location and time. Kot et al. 2022 showed, that the presence of technostress creators and inhibitors is crucial in shaping job satisfaction and work engagement [50]. The moderating effect of technostress inhibitors, particularly technical support, on the relationship between technostress creators and organizational commitment further underscores the complex interplay between these variables [52].

### Influence of preventive measures on technostress

The implementation of measures to prevent technostress remains crucial in order to avoid potential negative effects of the early use of technology, especially as the resources were not sufficiently accessible. In addition, the difference test carried out revealed significant differences in the level of technostress according to the degree of implementation of preventive measures, with the most significant differences between participants whose employers had already implemented a lot of measures and those whose employers had not implemented any measures at all. As a result, it was found that the group of doctors with a low level of implementation of preventive measures experienced higher levels of technostress. We can therefore verify assumption 4 in the light of these results. A systematic review of the literature on the outcomes of different interventions showed that a combination of different preventive measures could alleviate burnout symptoms caused by digitalization [53].

### Strengths and limitations

The design of our recruitment strategy, which was based on overview lists of the particular emergency departments and, as a result, ensured a complete selection and contact with the clinics, is one of the study’s strengths. The use of various validated and well-recognized scales, including the TAM model and the technostress scale by Ragu-Nathan et al., is a further strength. However, certain limitations of our study need to be addressed.

Due to the small number of study participants or the underrepresentation of residents and specialists in the study sample, it was not possible to conduct tests to analyse potential differences in technostress levels between the different groups. The recruitment strategy for surveys should therefore be improved to avoid the under-representation of certain groups of participants, such as residents, specialists or female doctors, as in the present study. For example, it might be an idea to focus on creating incentives for the respective subgroups to participate in studies such as this one.

In general, it should also be pointed out that the small sample size of our study and the underrepresentation of certain groups of participants, as described above, may limit the representativeness and thus the generalisability of our study results. Future studies on this topic should therefore include a larger number of participants. In addition, there is currently a lack of comparative studies from other medical specialities to relate the degree of technology use and correlations to the digital stress experience. Therefore, the available data cannot yet be adequately categorised.

### Implications for further research

Similar studies should be carried out in a longitudinal design to be able to track the evolution of technostress levels and potential changes over a longer time frame. Further investigation is also required because we still know very little about potential additional influencing factors that could have an impact on the levels of technostress experienced by doctors practicing emergency medicine. It would also be interesting to see whether there are differences in the perception of technostress between medical specialties depending on the type and frequency of use of digital technology. It is evident that there is a significant gap in the literature when it comes to comparative studies on technology use and digital stress in different medical specialties. Further research is needed to explore the varying impact of technology on healthcare professionals in fields such as cardiology, neurology, radiology, and various other specialties. Future studies could analyse the relationship between the extent of technology use and the extent of technostress and possibly show differences between different medical specialties, for example that doctors in emergency departments experience higher levels of technostress than other medical specialties due to their working environment.

By conducting comparative studies, researchers can gain a deeper understanding of how digital stress affects healthcare professionals across different specialties, ultimately leading to the development of targeted interventions and strategies. Additionally, these comparative studies can provide valuable insights into best practices for implementing technology in healthcare settings to minimize digital stress and maximize its benefits for both healthcare professionals and patients. Further exploration of this topic is crucial for the advancement of medical practice and the well-being of healthcare professionals.

Future studies could analyse the construct of technostress experience in a more differentiated way and shed light on the positive and negative sides of technostress and how to deal with it accordingly; for example, it could be analyzed under what circumstances technostress creators lead to eustress or distress in medical care based on the studies by Califf et al. (2015) [37].

To better understand the connections between technostress creators, resources, and various outcomes in this context, additional mediating variables should be taken into consideration. Additionally, there is still a dearth of knowledge about practical preventive measures and practical experience with their application. Therefore, more research is particularly required to advance our understanding of the preventive strategies that must be used to successfully counteract technostress. Field-based intervention studies looking at the effectiveness and satisfaction of various preventive measures are strongly suggested to close this research gap.

Since some studies have already suggested that participants with the aforementioned characteristics are more exposed to technology-induced stress and are at a higher risk of developing burnout, more research is necessary to determine whether these findings are consistent.

Future research on the variables used might consider additional mediating or influencing factors on technostress and the various outcomes, such as digital competence, self-efficacy, or training.

### Implications for practice

Some practical implications and potential solutions for the introduction and use of digital technologies in emergency medicine can be derived from the data obtained.

In order to cope with digital stressors and to reduce daily workload, emergency physicians can utilize various resources such as electronic medical record systems, telehealth technologies, and clinical decision support tools. These resources can help streamline communication and information management, allowing physicians to better prioritize tasks, make evidence-based decisions, and effectively manage their workload. Additionally, emergency physicians can also benefit from using secure messaging platforms specifically designed for healthcare professionals, as well as mobile apps that provide quick access to medical references and clinical guidelines [54]. By adopting these digital resources and employing strategies such as task prioritization, time management, and information organization, emergency physicians can mitigate the negative impact of digital stressors and improve their overall work efficiency and well-being [55]. Additionally, incorporating digital resources can also enhance collaboration and coordination among healthcare team members, leading to better patient outcomes [56]. It’s crucial for emergency physicians to stay updated on advances in digital tools and technologies that can support their clinical practice, as well as invest in ongoing training to maximize the use of these resources. Furthermore, establishing guidelines for digital communication and information sharing within the emergency department can help create a more structured and manageable digital environment [3, 57]. By proactively addressing digital stressors and harnessing digital resources, emergency physicians can navigate the demands of their profession more effectively, ultimately leading to improved patient care and physician satisfaction [54].

Clinics should invest in a robust and reliable technological infrastructure, carry out regular maintenance and updates and ensure adequate training of emergency physicians in the efficient use of the technology. It is also recommended that end users, e.g. doctors and nurses, are involved in the design and implementation process to ensure that the technology is in line with their daily clinical routines and meets their specific needs [58].

Close collaboration with the IT service team should be sought to understand concerns and provide the necessary support. Training programs should also be offered to improve their skills in using technology in healthcare [56]. Start with small pilot projects to demonstrate the feasibility and benefits of the technology. This can help build confidence among healthcare professionals, hospital management and IT teams. A dedicated support system should be set up to deal with any problems or concerns that arise during the implementation phase. This may include a helpdesk or dedicated team that can provide timely assistance to medical staff. The effectiveness of implemented technologies should be regularly evaluated and adjustments made based on feedback from healthcare professionals, patients and other stakeholders. This approach can help refine the usability and practicality of the technology over time [56].

Overall, addressing these challenges requires a collaborative effort involving healthcare professionals, hospital management, IT teams, and technology providers to ensure successful integration of new technologies into daily clinical routines in Emergency Medicine.

## Conclusions

In the field of emergency medicine, the integration of digital technologies has provided numerous benefits and advancements. However, there are also several challenges and problems that arise when using digital technologies in emergency medicine. One of the significant challenges that have been identified is the experience of digital stress among emergency physicians. It has been observed that the use of digital technologies, such as electronic health records and communication tools, can contribute to increased stress levels among physicians. However, this study shows that despite the diverse experience of digital stressors, the perceived level of technostress is moderate. Coping strategies and personality traits of the doctors could play a role here. While digital stress is still a concern in the field of emergency medicine, it is essential to address it through targeted interventions to support the well-being of physicians and optimize the use of digital technologies in patient care. To mitigate the impact of digital stress, it is crucial for healthcare institutions to implement strategies that promote digital well-being among emergency physicians. This may involve providing training on effective use of digital tools, ensuring user-friendly interfaces, and establishing support systems for physicians who may be struggling with the demands of digital technologies. There is a significant need for educational initiatives and training programs to increase awareness among healthcare providers in the emergency department setting. By addressing these knowledge gaps, emergency departments can better support patients and alleviate potential stressors related to digital technologies.

In light of recent scientific evidence and the results of the current study, the implementation of new digital technologies should be accompanied by strategic and quality-guided implementation of measures to effectively prevent digital stress, in order to make digitalization processes as effective as possible and thus to benefit from the full potential of hospital digitalization. In order to categorize and interpret the characteristics of the digital stress perception, a comparative study between different medical disciplines should be sought. In addition, future research should focus on intervention studies to learn more about the effectiveness of preventive measures.

### Electronic supplementary material

Below is the link to the electronic supplementary material.

**Additional file 1:** Contents of the online questionnaire

## Data Availability

The datasets analyzed during the current study are not publicly available due to German national data protection regulations but are available from the corresponding author on reasonable request.
