# Supplementary material for: Digital stressors and resources perceived by emergency physicians and associations to their digital stress perception, mental health, job satisfaction and work engagement
Source: BMC Emerg Med. 2024 Feb 27;24:31. doi: 10.1186/s12873-024-00950-x (PMC10900642; doi:10.1186/s12873-024-00950-x)
Supplement: Supplementary file 1 — Additional file 1: Contents of the online questionnaire [file 12873_2024_950_MOESM1_ESM.docx]

**Supplement**

Additional file 1: Contents of the online questionnaire

| **Section** | **Parameter** | **Instrument** | **Scale** | **Number of Items** |
| --- | --- | --- | --- | --- |
| A: Inclusion criteria | 1. Emergency medicine as a specialised discipline, 2. permanent employment as a doctor in emergency medicine (professional status/career level), 3. dealing with digital technologies (at least 1 group) | Self-developed | - | 3 |
| B: Personal and workplace details | 1.Age, 2. gender, 3. regional structure of the clinic (urban, small-town, rural), 4. organisation of the clinic, 5. duration of employment with employer, 6. weekly working hours, 7. total professional experience in total | Self-developed/  COPSOQ 2016 | - | 7 |
| C: Digital stressors in the workplace | Digital stressors in the workplace |  |  |  |
|  | 1. Overload due to digital technologies | Ragu-Nathan et al (2008)/ Gimpel et al (2018) | Overload | 5 (1-5) |
|  | 2. Complexity of digital technologies | Ragu-Nathan et al (2008)/ Gimpel et al (2018) | Complexity of digital technologies | 5 (12-16) |
|  | 3. Uncertainty in dealing with digital technologies | Ragu-Nathan et al (2008)/ Gimpel et al (2018) | Uncertainty in dealing with digital technologies | 4 (22-25) |
|  | 4. More specific stressors | HIMSS-Study (2015) | - | 11 (1-11) |
| D: Digital resources/ Stress-inhibiting factors | Stress-inhibiting factors |  |  |  |
|  | 1. Literacy facilitation | Ragu-Nathan et al (2008)/ Gimpel et al (2018) | Literacy facilitation | 5 (1-5) |
|  | 2. Involvement facilitation | Ragu-Nathan et al (2008)/ Gimpel et al (2018) | Involvement facilitation | 4 (10-13) |
| E: Preventive measures | 1. Information, qualification, participation | Bräutigam et al (2019) | Information before the introduction of new technology, qualification, participation | 7 (1-7) |
|  | 2. Detailed enquiry about prevention measures | HIMSS-Study (2015) | Prevention measures | 1 |
| F: Work-related health outcomes | 1. Burnout-Symptom | Copenhagen Burnout Inventory/ COPSOQ III (2022) | Burnout-Symptom | 3 (B13: 1-3) |
|  | 2. Job satisfaction | Ragu-Nathan et al (2008)/ Gimpel et al (2018) | Job satisfaction | 3 (I3: 1-3) |
|  | 3. Work engagement | COPSOQ III (2022) | Work enagement | 3 (B12:1-3) |
| G: Information on the frequency of use and evaluation of digital technologies as well as subjective benefits and usability | 1. Frequency of use of digital documentation technologies | Hübner et al (2018)/ Gensicke et al (2016) | Self-developed based on Hübner et al (2018) & Gensicke et al (2016); | 1 |
|  | 2. Period of use/proportion of time in a regular working day | HIMSS-Studie (2015) | Self-developed based on HIMSS study (2015) | 1 |
|  | 3. Subjective benefit & usability/usability of digital documentation technologies | (Holden, 2010) | Perceived Usefulness & Perceived Ease of Use | 11 (PU 1-6; PEOU 1-5) |
